# Back motion in unridden horses in walk, trot and canter on a circle

**DOI:** 10.1007/s11259-023-10132-y

**Published:** 2023-05-02

**Authors:** Agneta Egenvall, Hanna Engström, Anna Byström

**Affiliations:** 1 Department of Clinical Sciences, Faculty of Veterinary Medicine and Animal Science, Swedish University of Agricultural Sciences, Uppsala, Sweden; 2 Ekeskogs Riding Academy, Klintehamn, Sweden; 3 Department of Anatomy, Physiology and Biochemistry, Faculty of Veterinary Medicine and Animal Science, Swedish University of Agricultural Sciences, Uppsala, Sweden

**Keywords:** Kinematics, Optical motion capture, Lungeing, Circle, Horse, Back motion

## Abstract

Equine back function is of concern to riders, as well as to veterinarians and physiotherapists; these groups may benefit from knowledge about spinal motion on the circle. This descriptive and comparative study aimed to quantify equine neck, back and pelvic motion in walk, trot and canter on a 9 m circle. Sixteen healthy horses in training, of varying breed and conformation, were measured using optical motion capture (150 Hz), with optical markers on the poll, withers, T15, tubera coxae and lumbosacral joint. Cervicothoracic and thoracolumbar flexion–extension and lateral bending, and pelvic roll, pitch and yaw, were statistically evaluated using mixed models. Motion patterns showed distinct differences between gaits, but were generally similar between horses. The thoracolumbar back was bent towards the inside of the circle (stride mean 5-6º for all gaits). The cervicothoracic spine was more flexed in walk (18°), and more extended in canter (-4—-8°), compared to trot (6–7°), whereas the thoracolumbar spine was slightly less extended in canter than in walk. Thoracolumbar flexion–extension range of motion (ROM) increased from walk (4°) to canter (9°), as did pelvic pitch ROM (walk 7° and canter 15–16°), while back lateral bending ROM and pelvic yaw ROM were lowest in trot. Taken together, the study findings suggest that neck and back motion patterns on the circle reflect an interaction between the constraints of circular movement, and the mechanics and characteristics of each gait.

**Supplementary information:**

The online version contains supplementary material available at 10.1007/s11259-023-10132-y.

## Introduction

In quadrupeds, the back forms a functional connection between the limbs, and back motion is an integral part of the mammalian gait (Jones 2016). This makes equine back function of concern to riders, as well as to veterinarians and physiotherapists. Keeping the horse sound and symmetric are major goals for all these groups. When training horses, it is common to work the horse on a circle, both when ridden and from the ground. During lameness assessment, it is common to evaluate the horse trotting both straight and on the lunge, and for assessment of back function it is commonly recommended to do so in all three gaits and also while ridden (Davidson 2018). Studying equine back motion when the horse is moving straight, as well as when turning, is thus highly relevant both from a sports perspective and in clinical practice.

Back motion has been measured with optical motion capture, either using skin markers (Hardeman et al. 2020; Byström et al. 2021), or bone-fixed markers (Faber et al. 2000; 2001a; b), or with inertial measurement unit (IMU) systems (Greve and Dyson 2016; Greve et al. 2017; Martin et al. 2017; MacKechnie-Guire and Pfau 2021). While IMU systems show good agreement with optical motion capture (Pfau et al. 2005; Bosch et al. 2018), sensor displacement relative to the underlying bone can result in considerable inaccuracies at faster gaits, for example, doubling the estimated pelvic roll range of motion (ROM) in trot (Goff et al. 2010). Different studies have calculated back angles using either a horse-based coordinate system (Hardeman et al. 2020; Byström et al. 2021; MacKechnie-Guire and Pfau 2021) or the global or laboratory coordinate system (Faber et al. 2000; 2001a; b; Greve and Dyson 2016; Martin et al. 2016; Greve et al. 2017), as reference frame. In a majority of the latter studies, the angles measured are still referred to as flexion–extension, lateral bending and axial rotation, despite that the angles measured represent segment rotations (Euler angles) and not spinal joint motion. Consequently, angular values are often not directly comparable across studies. However, general conclusions on the expected pattern under various conditions can still be drawn from the available body of literature.

Equine back motion has been studied at walk (Faber et al. 2000), trot (Faber et al. 2001a; Hardeman et al. 2020; Byström et al. 2021; MacKechnie-Guire and Pfau 2021), and canter (Faber et al. 2001b; MacKechnie-Guire and Pfau 2021), both in unridden horses on a treadmill (Faber et al. 2000; 2001a; 2001b), and horses ridden on a straight line over ground (Greve and Dyson 2016; Martin et al. 2017; MacKechnie-Guire and Pfau 2021). In unridden horses, flexion–extension ROM was smallest in trot, larger in walk and largest in canter for most segments, whereas lateral bending ROM was relatively similar across gaits (Faber et al. 2000; 2001a; b). Comparing ridden trot and canter (MacKechnie-Guire and Pfau 2021), T18-L3 flexion–extension (‘differential pitch’) ROM and T13-sacrum lateral bending (‘differential heading’) ROM were larger in trot. Back movements on circles has only been studied in unridden horses in trot (Greve et al. 2017; Hardeman et al. 2020; Byström et al. 2021). The thoracolumbar back showed a 4° increase in lateral bending towards the inside of the circle compared to straight (on average during the stride), and the angle between the neck and the body increased by 5°, indicating that the horses turned the head towards the circle centre (Byström et al. 2021). Further, back lateral bending ROM tended to increase on the circle (Greve et al. 2017; Byström et al. 2021).

In summary, while both training and clinical evaluation of horses on circles are very common, equine back motion on the circle has not been studied in walk or canter, whereas studies of horses moving in a straight line have shown that back motion differs between gaits. Determination of angular back motion of normal horses on the circle may serve as bases for further research on, for example, back problems or effects of physiotherapy interventions, similar to such studies already conducted for straight-line motion and/or trot only (Faber et al. 2003; Wennerstrand et al. 2004; Spoormakers et al. 2023 in press). The aim of the current descriptive and comparative study was to quantify ROM and mean by stride for cervicothoracic, thoracolumbar, and pelvic angles in unridden horses while moving on a ~ 9 m diameter circle in walk, trot and canter in both left and right directions, in order to reveal systematic patterns and differences between gaits. It was hypothesised that neck and back movements would differ between gaits, and between left and right directions for lateral bending mean. It was anticipated that differences between gaits would be similar to findings for straight line for flexion–extension, but differ for lateral bending, given that lateral bending differs more between straight line and circle in trot.

## Materials and methods

### Horses

Sixteen horses were used in the study. They comprised 6 mares, 8 geldings and 2 stallions, all boarded at the riding establishment where the data collection took place. Horses’ age ranged between 4 and 24 years, median 11 years. A variety of breeds and sizes were represented. Several horses were Iberian, or Iberian cross. The smallest horse was a pony and the tallest a Swedish warmblood (see SI 1 Table). All horses were unshod. One horse had not yet been trained under saddle, but had been educated in groundwork for a year. The remaining horses were educated in classical dressage to varying levels; none of the horses were used for competition. All horses were in active work, and were considered sound by their owners. During data collection, the horses were assessed by a veterinarian (author AE) while moving in hand and on the lunge on soft surface, and deemed to be sound in walk and trot and to have normal back function while moving; according to palpation, visual assessment of the movement pattern, and based on the owners’ perception. According to Swedish law, ethical approval is not required for non-invasive experiments that do not expose animals to any risks above their normal daily activities. Horse owners gave written informed consent for the data collection.

### Markers

**Fig. 1:**
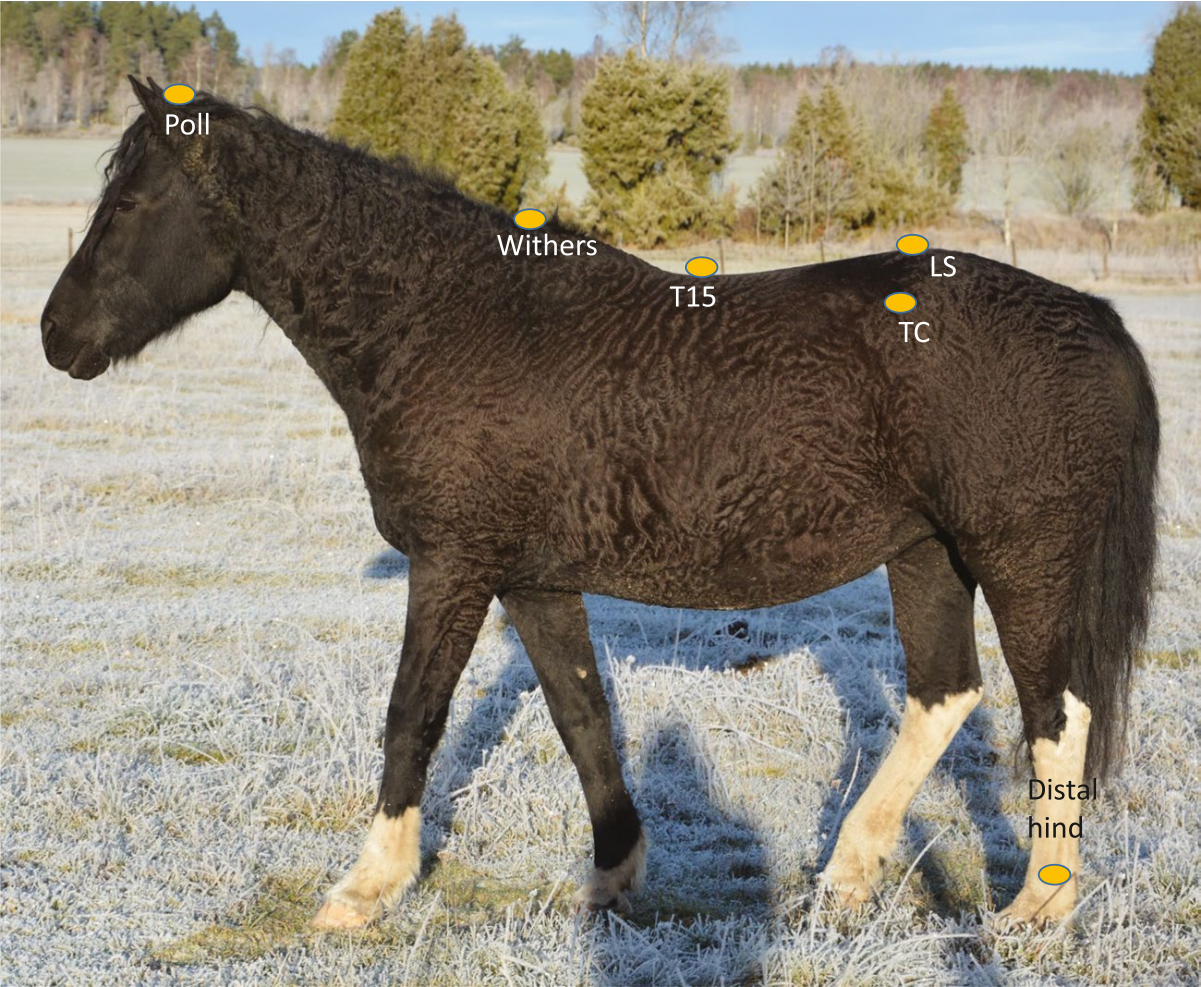
Marker placement. The markers used in the current study were placed at the poll, the highest point of the withers (T6), the spinous process of the 15^th^ thoracic vertebra (T15), at the lumbosacral joint (LS), left and right tubera coxae (TC), and over the laterodistal part of the third metatarsal bones

Spherical retroreflective markers of 25 mm diameter were attached with double-adhesive tape. The markers used in this study were placed at the poll, the highest point of the withers (T6), the spinous process of the 15^th^ thoracic vertebra (T15), over the lumbosacral joint, left and right tubera coxae, and over the laterodistal part of the third metatarsal bone (MTIII). Marker placement is illustrated in Fig. 1.

### Data collection

Optical motion capture took place in an indoor arena (20*30 m, SI 2 Fig.). The arena surface consisted of a sand and synthetic fibre mix. The measuring volume was approximately 10 × 10 × 3 m, the maximal volume that could be sufficiently covered by 12 high-speed infrared cameras (Oqus 700 + a, Qualisys AB, 411 05 Gothenburg, Sweden) available. The camera system was set to record at a sampling frequency of 150 Hz. Three cameras were placed on tripods, the remaining were wall-mounted 2–3 m above the ground (SI 2 Fig.). The perimeter of the volume was marked with ground poles, to guide the horses and handlers. Data collection took place after dusk to avoid sunlight interfering with the motion capture (at an ambient temperature of -5 to + 5 °C). Calibration of the camera system was performed daily before the first measurement and was accepted only if the average calibration residual was below 3.5 mm, otherwise the calibration was repeated. The experiments were video-recorded (Sony FDR-AX53, 25 Hz) for documentation.

Following marker placement, horses performed the following exercises (only data for the lungeing exercises were used in this study): walk in-hand on a straight line on the diagonal through the calibrated volume, at least 2 times, then walk in hand two full circles to the left and to the right, lungeing for two full ~ 9 m diameter circles in left and right directions first in walk, then in trot, and then in canter. The horses were handled by their owner in most cases, otherwise a person with which the horse was familiar (n = 6 different handlers). The handler strived to keep the same circle radius in all gaits, guided by the ground poles enclosing the measurement area. Speed was selected by the handler such that the horse looked as comfortable as possible in each gait, and care was taken to achieve similar speeds in both directions. Warm-up before the above described protocol was minimal, but all horses had been turned out during the day and came in from the field shortly before the measurements. One or two horses were measured each data collection day. Initial lungeing direction, left or right, was alternated between horses, nine horses started to the left and seven to the right.

### Data analysis

Kinematic data were analysed in Matlab (version R2020a) using custom-written scripts. The following variables were calculated from the optical motion capture data: Speed was determined from the movement of the lumbosacral joint marker in the horizontal plane. Pelvic roll (angular rotation in the frontal plane) was calculated using the tuber coxae markers. Pelvic pitch (angular rotation in the sagittal plane) was calculated using the marker at the lumbosacral joint and the average of the two tuber coxae markers. Pelvic yaw (angular rotation in the dorsal plane) was calculated using the two tuber coxae markers. Pelvic roll was determined relative to the horizontal, while pitch and yaw were determined from the cross product with a line between the withers and lumbosacral markers to account for orientation of the horse’s body. Pelvic roll angle was used to approximate body lean of the horse. Before calculating cervicothoracic and thoracolumbar flexion–extension and lateral bending angles the coordinate system was adjusted accordingly (based on the pelvic roll average in a moving window of length stride duration times sampling rate, centred on the data frame in question). This was done in order for the x–y and the y–z planes to correspond as closely as possible to the dorsal and the sagittal planes of the horse, respectively, to avoid projection errors. Flexion–extension and lateral bending angles for the thoracolumbar back were then determined between the markers at the highest point of the withers, T15 and at the lumbosacral joint, in the sagittal plane for flexion–extension and in the dorsal plane for lateral bending. Cervicothoracic flexion–extension and lateral bending angles were likewise determined, using the markers on the poll, the withers and T15. The trunk horizontal angle (also called body tracking, Hardeman et al. 2020), representing the orientation of the horse’s body (a line between the withers and lumbosacral joint markers in the horizontal, i.e. x–y plane), was calculated relative to the direction of movement (velocity vector, i.e. [dx, dy]) for the midpoint between the withers and lumbosacral joint markers. The neck-to-trunk angle, representing the head position relative to the body (also called head swivel), was calculated as the angle in the horizontal plane between the horse’s body (as above) and the neck (a line between the head and withers markers). Neither of these two angles were corrected for body lean for these angles to be directly comparable to previous studies. (Pelvic pitch and yaw are adjusted for body lean inherent to how these angles were calculated.)

**Fig. 2:**
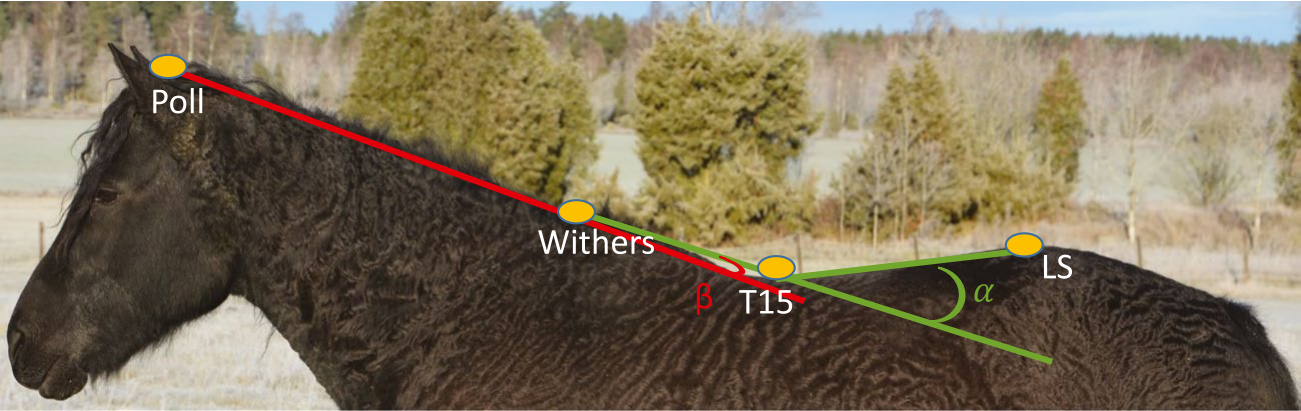
Thoracolumbar (α) and cervicothoracic (β) flexion–extension angle definitions. Lines between utilised markers are inserted to illustrate the angles. The angular value is defined as zero if the three markers were at equal height, positive for flexion and negative for extension. In this illustration both the cervicothoracic angle (β) and the thoracolumbar back (α) flexion–extension angles are in extension, although the cervicothoracic angle only slightly so. T15 – 15^th^ thoracic vertebra, LS –lumbosacral joint

**Fig. 3:**
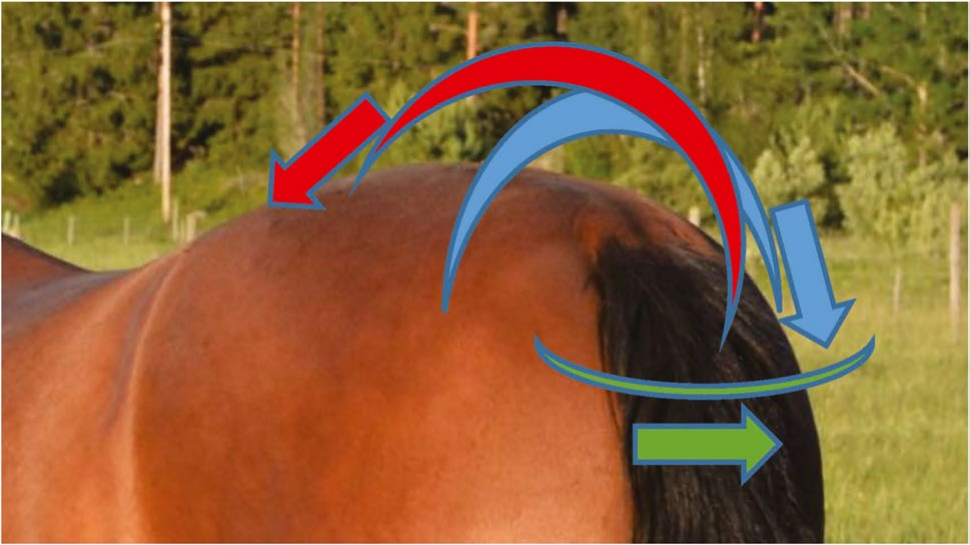
Pelvic rotations. Arrows indicate positive directions. Pelvic roll (blue) was defined as positive for clockwise rotation seen from behind, positive roll corresponding to a relative lowering of the right tuber coxae. Positive pitch (red) was defined as clockwise rotation seen from the right, corresponding to relative upwards movement of the dock of tail. Positive yaw (green) was defined as counter-clockwise rotation seen from a dorsal view, corresponding to movement of the tail towards the right

Flexion–extension angles were defined as zero if the three markers were at equal height, positive for flexion and negative for extension (Fig. 2). Lateral bending angles were defined as zero when the three markers were aligned in the sagittal plane, negative for bending to the left and positive for bending to the right. Pelvic roll was defined as positive for clockwise rotation seen from behind, corresponding to a relative lowering of the right tuber coxae; pitch was defined as positive for clockwise rotation seen from the right, corresponding to relative upwards movement of the dock of tail; yaw was defined as positive for counter-clockwise rotation seen from a dorsal view, corresponding to movement of the tail towards the right. Pelvic rotations are illustrated in Fig. 3. The trunk horizontal angle was defined as positive for clockwise rotation from a dorsal view (‘forehand to the right—hindquarters to the left’ deviation) relative to the direction of motion. The neck-to-trunk angle was defined as positive if the head was to the right of the body axis.

Time-series traces for the above variables were segmented into strides based on maximum protraction of the inner hind limb. Hind limb protraction-retraction angle was calculated between a line from the withers’ marker to the lumbosacral joint marker, and a line from the lumbosacral marker to the laterodistal third metatarsal bone (MTIII) marker. Protraction-retraction angle data were band-pass filtered using a zero-lag Butterworth filter with cut-offs at 0.5 and 4 times the stride frequency.

For each stride, mean and ROM were calculated for all of the above-mentioned variables (other than hind limb protraction-retraction, which was solely used for stride splitting). The only exceptions were that ROM was not calculated for neck-to-trunk and trunk horizontal angles. Circle radius was determined for each measurement (trial) through fitting a circle to the x and y (horizontal plane) coordinate data from the lumbosacral joint marker using the least squares method. Expected body lean was calculated from circle radius and speed using the following formula: tan^−1^(speed^^2^/circle radius * 9.81, Pfau et al. 2012), with the assumption that variations in circle radius throughout a measurement were small enough to be neglected. The difference between expected body lean and stride mean pelvic roll was then calculated.

### Statistical analysis

Stride curves were time normalised to stride duration and plotted as trial means and standard deviations (using Matlab). Descriptive statistics were tabulated. Mixed models were constructed (SAS version 9.4, PROC MIXED) to analyse differences between gaits, and between left and right directions in each gait, for the above described kinematic variables (used as outcome variables).

Speed was modelled as a linear variable as speed was assumed to have linear relationships to the outcome variables. This was considered a reasonable approximation given that the speed range recorded for each horse and gait was small.

For all dorsal- and frontal plane variables, corresponding movements, e.g. lateral bending to the inside of the circle, have opposite signs for left versus right direction. This means that any circle-induced effects on stride mean will also have opposite sign between directions. These variables comprise neck-to-trunk angle mean, cervicothoracic lateral bending mean, thoracolumbar lateral bending mean, pelvic roll mean, pelvic yaw mean and trunk horizontal angle mean. For mean (but not ROM) for these variables, speed was therefore nested within direction when included in the models. For mean for sagittal plane variables, i.e. flexion–extension and pelvic pitch, and for ROM for all variables, speed was modelled without nesting. Model formulas (detailed below) were otherwise the same. Random effects were horse and the interaction between direction and horse. In a few models horse was omitted because the G-matrix was not positive definite when horse was included, leaving horse nested in direction in these cases.

Mixed models were first made stratified by gait. These models included direction and speed as fixed effects. Models were then made with data for all three gaits, to investigate differences between gaits. These models included gait, direction, and the interaction between gait and direction as fixed effects. During preliminary modelling, speed was also included. However, this required a three-way interaction with gait and direction for stride mean for dorsal- and frontal plane variables, which made model results difficult to interpret. Since speed variations were relatively small for each horse and gait, speed was therefore omitted. Least square means for each gait and direction were compared to the results from the speed-corrected gait-specific models to confirm that this was a reasonable decision. Circle radius was likewise included in preliminary models, but this produced unstable and inconsistent results, likely because circle radius was calculated per trial and thus had zero within-trial variation. Circle radius was therefore omitted.

Finally, a model was made with the difference between stride mean pelvic roll and expected body lean as outcome variable, using data for trot and canter only (the horses did not show body lean in walk as deduced from stride mean pelvic roll). Direction and gait and their interaction were included as fixed effects. Speed was omitted, since it is included in the formula for calculating expected body lean.

Alpha was set to 0.05 in all analyses. Fixed effects were reduced until global (type III) p-values for all remaining variables had p < 0.05, after which pair-wise comparisons were performed using the SAS-option pdiff. P-values were not corrected for multiple comparisons. Residuals were scrutinised for normality, using scatter plots and QQ-plots. If residual distributions were judged suboptimal, transformation along the ladder of powers was attempted using Box-Cox transformation. Results from Box-Cox transformation suggested that all variables were the closest to normality when untransformed and were modelled untransformed accordingly. The residual pattern was suboptimal for thoracolumbar lateral bending mean and pelvic roll mean, but was not improved through transformation. For pelvic pitch mean square root transformation was tried, but model results were almost identical (data not shown) compared to when untransformed. Data and code for the statistical analysis, along with full model printouts, can be found at: https://figshare.com/s/e64a3afb518d4809f9b0.

## Results

### General results

**Fig. 4:**
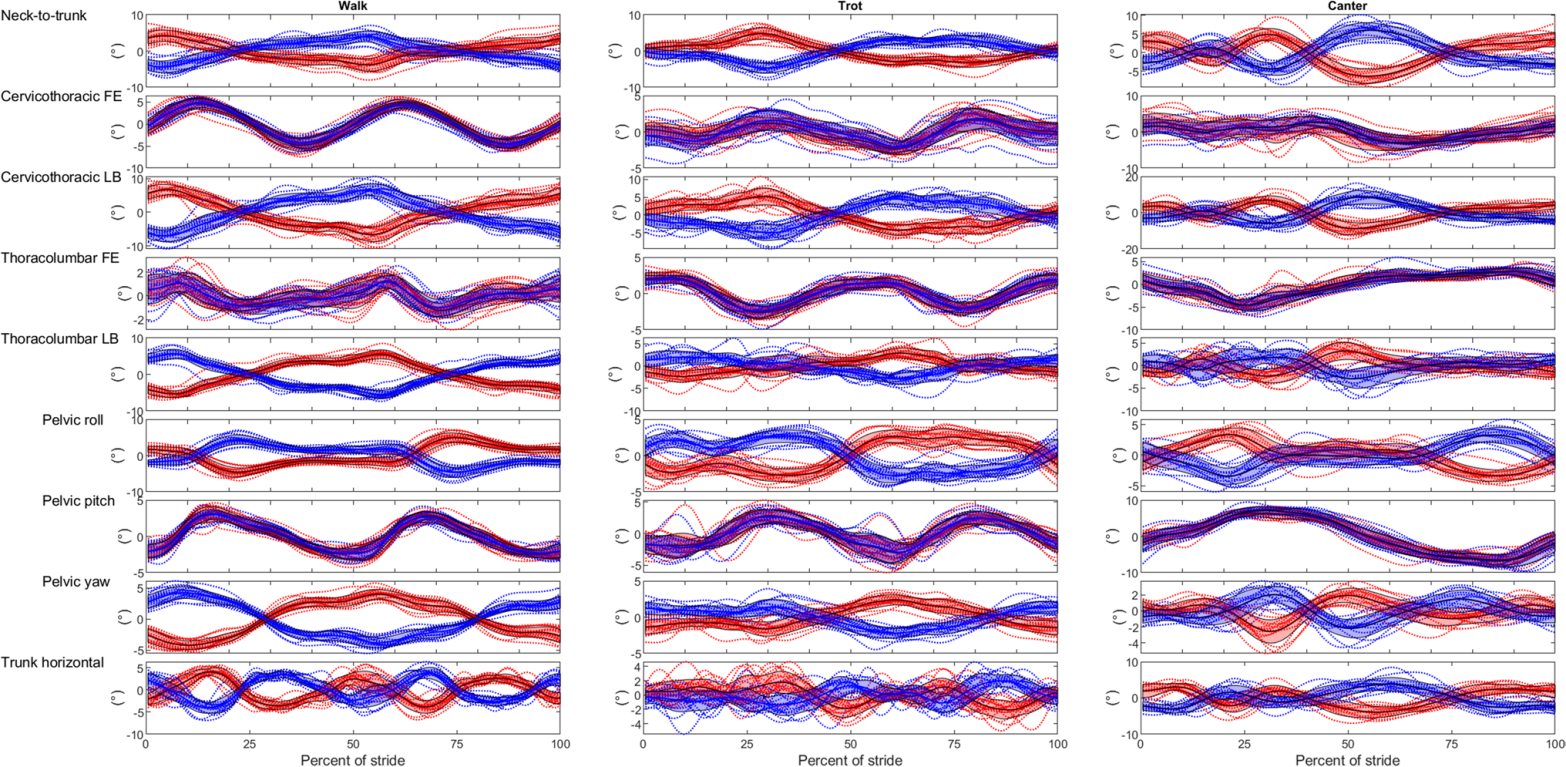
Time-normalised stride data by direction: left (red) and right (blue) in walk, trot and canter for all 16 horses. The x-scale is in percent of the stride relative to inside hind limb maximum protraction. Interrupted lines represent horse and direction means. Black lines represent mean values across horses, and the surrounding shaded area SD. Lateral bending angles were defined as negative for bending to the left and positive for bending to the right. For definition of pelvic rotations see Fig. 3. The trunk horizontal angle was defined as positive for ‘forehand to the right—hind quarters to the left’ deviation relative to the direction of motion, and the neck-to trunk angle was positive if the head was placed to the right relative to the orientation of the body

The full dataset, with data from 16 horses, contained 1636 strides. There were 651 strides in walk, 654 in trot and 331 in canter. Only 13 horses had data for canter (two horses did not manage to canter in both directions on the small circle required, and data for one horse could not be used due to poor data quality for the markers used for stride splitting). There was some additional dataloss for some variables due to missing marker data because of suboptimal camera coverage (see Tables 1 and 2 for the exact number of strides included in each analysis). Figure 4 shows time-normalised data for all horses for each of the variables analysed. Least square means from the models stratified by gait (with speed included) were generally very similar (SI 3 sheets ‘sagittal models’ and ‘dorsal and frontal’, note that model results are lacking if all fixed effects had p ≥ 0.05) to those from models including data for all gaits (without speed, Tables 1 and 2).

**Table 1:**
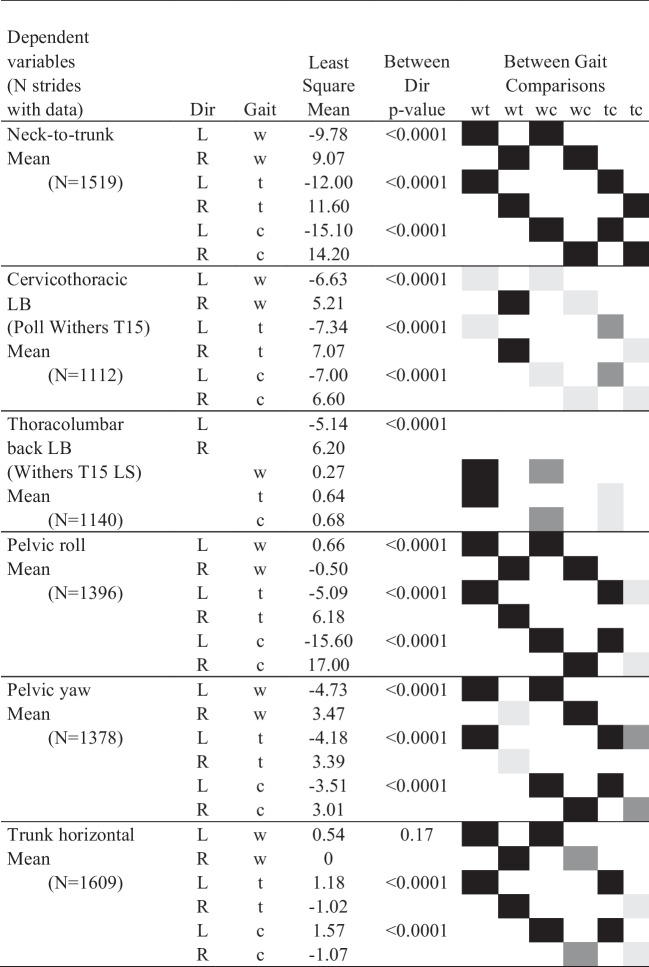
Least square means (in °) by gait and direction for frontal and dorsal plane variables, and contrast p-values for comparisons between directions (Dir) and between gaits. Grey and black squares show pairwise comparisons performed between gaits. Significant difference between gaits are indicated with pairs of shaded squares in the right-most columns, black represent *p* < 0.0001, dark grey *p* < 0.05 and ≥ 0.0001, and light grey *p* ≥ 0.05

Lateral bending angles is negative for bending to the left and positive for bending to the right. For definition of pelvic rotations, see Fig. 3. Trunk horizontal angle is positive for ‘forehand to the right—hindquarters to the left’ deviation relative to the horse’s direction of motion. Neck-to trunk angle is positive if the head is to the right relative to the orientation of the body

w: walk, t: trot, c: canter, LB: lateral bending, LS: lumbosacral joint, wt comparison walk—trot, wc comparison walk—canter and tc comparison trot – canter

**Table 2:**
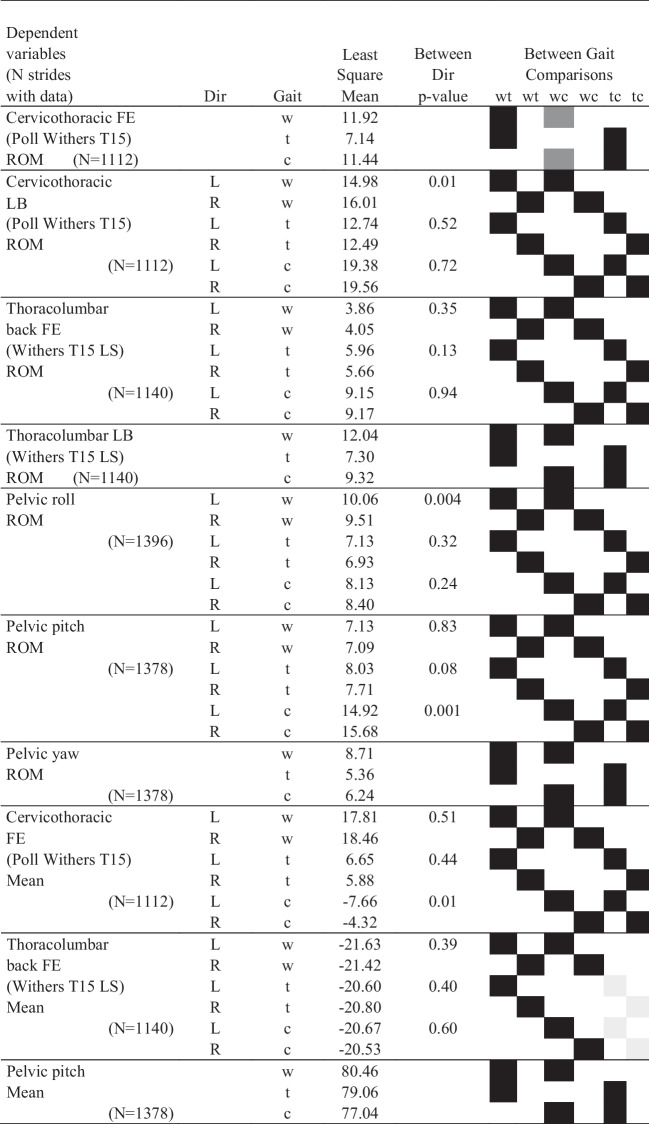
Least square means (in °) by gait and direction for mean for sagittal plane variables and range of motion (ROM) for all analysed variables, including contrast p-values for comparisons between directions (Dir) within gait. Grey and black squares show pairwise comparisons performed between gaits. The two-way interaction between direction and gait was significant (p < 0.05) for all variables, except cervicothoracic flexion–extension, thoracolumbar lateral bending, pelvic yaw ROM and pelvic pitch mean. Significant difference between gaits are indicated with pairs of shaded squares in the right-most columns, black represent *p* < 0.0001, dark grey *p* < 0.05 and ≥ 0.0001, and light grey *p* ≥ 0.05

Flexion–extension is positive for flexion and negative for extension, for definition of pelvic rotations see Fig. 3

w: walk, t: trot, c: canter, FE: flexion–extension, LB: lateral bending, LS: lumbosacral joint, wt comparison walk—trot, wc comparison walk—canter and tc comparison trot – canter

Descriptive statistics for all variables, by gait and direction, can be found in supplementary information 1 (SI 3 sheet ‘descriptive’). Trial mean speed was lowest in walk (1.2 m/s), higher in trot (2.4 m/s), and highest in canter (3.7 m/s).

### Mean angles for cervicothoracic and thoracolumbar lateral bending, neck-to-trunk angle, and trunk horizontal angle

In the models including data for all gaits, direction*gait interaction was significant for stride mean cervicothoracic lateral bending, neck-to-trunk and trunk horizontal angles, whereas it was not for thoracolumbar lateral bending (Table 1). Least square means suggest that the horses bent to the inside in both directions (lateral bending is negative for bending to the left, positive to the right, Table 1). Results for cervicothoracic lateral bending and for the neck-to-trunk angle suggest similar conclusions, but estimates differ in magnitude, and there were fewer significant differences between gaits for cervicothoracic lateral bending versus for the neck-to-trunk angle.

### Mean angles for cervicothoracic and thoracolumbar flexion–extension

The gait*direction interaction was significant for both cervicothoracic and thoracolumbar mean flexion–extension (Table 2). All between-gait comparisons were significant, except comparing thoracolumbar flexion–extension mean between trot and canter (borderline for the left direction). Least square means for cervicothoracic flexion–extension mean indicated extension in canter, mild flexion in the trot and relatively more flexion in the walk (Table 2, SI 3 sheet ‘dorsal- and frontal’). This is opposite to thoracolumbar flexion–extension mean, which was slightly more extended in walk (left -21.6º, right -21.4º) compared to trot (left -20,6º, right -20.8º) and canter (left -20.7º, right -20.5º).

### Mean pelvic roll, pitch, and yaw angles per stride

The gait*direction interaction was significant for stride mean pelvic roll and yaw mean (Table 1), whereas only gait was significant for pelvic pitch mean (Table 2). Pelvic pitch mean was largest in walk (80°) and smallest in canter (77°). Results for pelvic yaw indicate that the pelvis was rotated with the tail towards the inside in both directions in all gaits (Table 1), but slightly more so in walk (left -4.7°, right 3.5°) and least in canter (left -3.5°, right 3.0°). Pelvic roll mean (Table 1) was near zero in walk, larger in trot (5–6°) and largest in canter (16–17°). For the difference between pelvic roll mean and expected body lean there where was no effect of direction (n = 824 trot and canter strides in this model). However, there was a difference between gaits (p < 0.0001): in canter the horses leaned 3.4° less than expected, which was a bigger difference than in trot where they leaned 2.8° less than expected.

### ROM variables

In the models including data for all gaits, the direction*gait interaction was significant for four of seven ROM variables, and for these variables gaits differed significantly in all comparisons (Table 2). Thoracolumbar flexion–extension ROM was smallest in walk (~ 4°), while thoracolumbar lateral bending ROM (15–16°), and pelvic roll (~ 10°) and yaw ROM (~ 9°), were all largest in walk. Cervicothoracic flexion–extension ROM (6–7°), thoracolumbar lateral bending ROM (7°) and pelvic yaw ROM (5°) were smallest at the trot. Cervicothoracic lateral bending ROM (19–20°) and pelvic pitch ROM (15–16°) were both largest in canter. Pairwise comparisons between directions within gait were significant for cervicothoracic lateral bending and pelvic roll ROM in walk, and pelvic pitch ROM in canter. In the models stratified by gait, where speed was included, only thoracolumbar flexion–extension ROM in trot differed significantly (p = 0.03) between directions: ROM was 6.0° in the left direction and 5.6° in the right direction.

### The effect of speed

Speed was only included in the gait-specific models. For dorsal- and frontal plane mean angle variables speed was modelled nested in direction (SI 3 sheet ‘dorsal and frontal’). A significant speed effect was found for five of these variables in at least one gait. The estimates for speed have opposite signs between directions, as expected. For example, for the neck-to-trunk angle in walk, the speed estimate is positive for left direction and negative for right direction, suggesting that the head was placed less to the inside relative to the orientation of the body with increasing speed in both directions. For sagittal plane mean angle variables and all ROM variables speed was modelled as a simple linear effect without nesting (SI 3 sheet ‘sagittal’). A significant speed effect was found in at least one gait for all these variables. For example, the positive speed coefficient for cervicothoracic flexion–extension ROM in walk suggests that ROM increased with increasing speed (disregarding direction).

### Random term contribution

Random effect loadings vary between models (SI 3 sheet ‘random’). For example, comparing models with data for all gaits, the neck-to-trunk angle mean model had a large residual variation (unexplained random variation, 52% of the total random variation in the model), while for thoracolumbar flexion–extension mean and for pelvic pitch mean the majority of the random variation was attributed to horse (89% and 99%, respectively). Similarly, in the models stratified by gait over 90% of the variation was attributed to horse for thoracolumbar flexion–extension mean and pelvic pitch mean. The direction*horse interaction generally contributed the most in models for dorsal- and frontal plane mean angle variables. The largest relative contribution was found in the (gait-specific) model for trunk horizontal angle in canter (85%).

### Individual patterns

SI 4 Figs. 1–2 show stride curves for cervicothoracic, thoracolumbar and pelvic angles in walk, trot and canter for two example horses (one Swedish Warmblood, horse D, SI 4 Fig. 1 and one Irish Cob, horse S, SI 4 Fig. 2).

## Discussion

The current study compared back and pelvic motion on the circle in unridden horses across the horse’s basic gaits, walk, trot and canter. Contrary to our hypothesis, the findings suggest that horses show a relatively similar degree of cervicothoracic and thoracolumbar lateral bending to the inside in all three gaits when moving on a relatively small (9 m diameter) circle. However, in accordance with our hypothesis, clear differences between gaits were observed in cervicothoracic and thoracolumbar lateral bending ROM and pelvic yaw ROM, with higher values for walk and canter than for trot. Earlier studies describing thoracolumbar back motion in unridden horses in straight line or on treadmill (Faber et al. 2000; 2001a; b; Haussler et al. 2001; Johnston et al. 2002; 2004) have found lateral bending ROM to differ the least between gaits, both based on back segment rotations and relative angles between segments. This discrepancy is in accordance with our hypothesis, we expected gait effects and circle effects to interact the most for lateral bending. That trot showed the lowest ROM likely relates to the necessity of stabilising against twisting forces induced by the diagonal stance, as reflected by trunk muscle activity (Kienapfel et al. 2018). In unridden horses moving straight, smaller ROM in trot than in walk or canter has been observed for thoracolumbar flexion–extension and axial rotation (Faber et al. 2000; 2001a; b; Haussler et al. 2001). In the current study, thoracolumbar flexion–extension and pelvic pitch ROM showed a progressive increase from walk to canter. However, cervicothoracic flexion–extension ROM was larger in both walk (12°) and canter (11°) compared to trot (7°). A large cervicothoracic flexion–extension ROM is inherent to the walk, where coordinated flexion–extension of the neck is used as an energy conserving mechanism through elastic recoil and collision mechanics (Gellman and Bertram 2002; Loscher et al. 2016). Collision mechanics has also been used to describe energy conservation strategies in canter (Ruina et al. 2005) and timed neck movements might provide benefits similar to those in walk. These findings confirm our hypothesis that neck and back motion patterns on the circle are the result of an interaction between the constraints of circular movement, and the mechanics and characteristics of each gait. A previous study in unridden horses comparing temporal characteristics and limb posture between walk, trot and canter on the circle similarly concluded that adaptation to curved movement is gait-specific (Hobbs et al. 2011).

The speed of motion is one possible reason for differences between gaits in adaptation to circular movement. The lateral force needed for turning is proportional to speed squared; for example, if speed is doubled, the force required to maintain the same turn radius increases four times. If the resultant vector between the vertical and lateral ground reaction forces points to the inside of the centre of mass, it will act to tip the horse over to the outside (Hobbs et al. 2011). This makes it challenging for the horse to turn on a small circle at high speed, and horses generally lean into the circle proportionally to the speed to counteract this effect (Hobbs et al. 2011; Pfau et al. 2012). Body lean has previously been approximated as pelvic roll mean (Pfau et al. 2012; Brocklehurst et al. 2014; Greve and Dyson 2016; Greve et al. 2018; Byström et al. 2021). In the current study, pelvic roll mean was near zero in walk, 5–6° in trot, and around 16° in canter. This suggests that the average study horse leaned into the circle about twice as much in canter compared to trot, and was upright in walk. In addition to trunk lean, the horse may theoretically also use the head-neck segment to balance. The results for the neck-to-trunk angle suggest that the horses positioned the head more to the inside relative to the body with increasing speed (walk 9°, trot 12°, canter 14–15°). However, cervicothoracic (poll—withers—T15) lateral bending mean differed only marginally between gaits. This may relate to that the neck-to-trunk angle was not corrected for body lean. The neck-to-trunk angle could thereby be affected by projection errors at faster gaits. However, if the horse tilted the hindquarters more into the circle compared to the forehand, or kept the neck more upright compared to the body, it is also possible that the cervicothoracic angle became somewhat overcorrected at faster gaits, and that the truth lies somewhere in between. Even though pelvic roll has been assumed in several studies to be representative for body lean (Pfau et al. 2012; Brocklehurst et al. 2014; Greve and Dyson 2016; Greve et al. 2018; Byström et al. 2021), this has not yet been verified by means other than that the results are plausible based on the expected degree of body lean calculated from speed and circle radius (Pfau et al. 2012). Future studies should investigate the absolute and relative orientation of the horse’s body segments on the circle in more detail, including individual variation in these adaptations.

For trot and canter, values for pelvic roll mean in the current study 5–6° and around 16°, respectively, are smaller, i.e. indicating less body lean, compared to previous studies of unridden horses (Pfau et al. 2012; Brocklehurst et al. 2014; Byström et al. 2021). The values are also smaller than reported values for body inclination in unridden horses based on the angle between the sacrum and the distal hind limb (5.3° at walk, 18.8° at trot and 24.8° at the canter, Hobbs et al. 2011). However, speeds were also lower in the current study. Given the relationship between speed and body lean (Pfau et al. 2012), it is more relevant to compare the divergence from the expected lean angle based on speed and circle radius (as described in Pfau et al. 2012). The horses in the current study leaned 2.8° less than expected in trot and 3.4° less than expected in canter. In the study by Pfau et al. (2012), where dressage horses were lunged in trot on circles with diameters ranging from 4–22 m, horses were leaning 1.2° less than expected on average. However, values for individual horses ranged between -8.1–3.8°. All horses in the current study had been regularly trained in work on the lunge on circles of different sizes. This may have contributed to why they leaned less, as Greve and Dyson (2016) found that well-trained horses lean less on the circle than those less well trained, both when ridden and on the lunge. Still, several of the horses found it relatively challenging to canter on a 9 m circle. This resulted in fewer canter strides (or no data) for some horses (circle size could not be increased due to limitations of the camera setup). In contrast, all horses appeared quite comfortable at walk and trot. The cervicothoracic flexion–extension mean angle changed from moderate flexion (around 18°) in walk and mild flexion in trot (6–7°) to mild extension (-4 to -6°) in canter, which may reflect a more relaxed posture in the slower gaits. Concurrently the thoracolumbar back became slightly more flexed and the pelvis more tilted backwards (indicating lumbosacral flexion) from walk to canter. The combination of increased thoracolumbar flexion and increased cervicothoracic extension is somewhat surprising given the coupling between cervicothoracic and thoracolumbar extension previously observed in cadaveric studies (Denoix 1999), as well as in live ridden horses (Rhodin et al. 2009). This discrepancy may be due to the constraints of the circle, or that top line posture was compared between gaits rather than between different head-neck positions in the same gait. It should also be noted that the difference in mean cervicothoracic angle from walk to canter was ten times larger than the difference in mean thoracolumbar angle.

Horses show individual variation in their adaptation to circular movements, as well as in spinal kinematics. Previous studies of unridden horses have found differences in body lean during trot on a circle between horses and between left and right directions (Brocklehurst et al. 2014), and differences in back ROM related to conformation in walk and trot on a treadmill (Johnston et al. 2002). Individual variation in cervicothoracic, thoracolumbar and pelvic ROM and stride patterns can be appreciated from Fig. 4 and Fig. SI 4. The current group of horses is too small to determine reasons for these variations or categorize the horses into subgroups, but breed/conformation seems the most influential factor, whereas there was no obvious relation to horse age. At group level, thoracolumbar flexion–extension ROM was slightly higher trotting in left versus right direction, i.e. horses were slightly more movable going left. It can further be noted that angle mean values for the left direction were often somewhat larger in absolute terms than those for the right direction. This may reflect underlying population-level asymmetry or side preference, i.e., laterality (Byström et al. 2020), similar to handedness in humans. However, small differences in mean lateral bending and pelvic roll and yaw between directions should be interpreted with great care due to the possibility of small asymmetries in marker placement. Symmetric marker placement is a challenge with skin-mounted markers. However, ROM is generally less affected by asymmetric marker placement compared to angular min, max and mean values (Audigié et al. 1998). Further, the similarities between horses across gaits outweigh the individual variations by far, despite that the current study group was rather diverse, in terms of both size and breed. The findings suggest that horses show similar spinal movement patterns overall in each gait, despite differences in confirmation and withers height.

Comparing speed estimates for ROM variables in trot in the current study to those in a previous study in unridden horses trotting on circles (that were significant in at least one direction, Byström et al. (2021)), findings agree for thoracolumbar flexion–extension, thoracolumbar lateral bending, and pelvic pitch and yaw, and were relatively similar for pelvic roll (somewhat smaller estimate in the present study). In the present study speed coefficients for pelvic roll ROM were positive for walk and canter, but negative for trot. This is in line with the general differences between gaits, with more spinal motion in walk and canter compared to trot. For pelvic pitch mean, the speed coefficients were positive for the three gaits, suggesting that with increasing speed the pelvis tilts more forward in all three gaits. However, since the range of speeds recorded for each horse in the current study was small, the estimated speed effects should be interpreted with care.

### Benefits and limitations

The study population was diverse, which is both a limitation and a benefit, making applicability of the results to a wider horse population more likely. The number of horses was limited and canter data were missing for a few horses. Due to the small measurement volume, limited by the number of cameras available, it was only possible to measure the horses on a single circle size, which was relatively small. However, horses were accustomed to working on circles of different sizes and were able to maintain the circle size without excessive influence from the handler. Ideally we would have liked to have measured the horses on a 10 or 12 m circle as well, since somewhat larger circle sizes is more common in practice and in previous studies. The current study addressed general movements within the cervical and thoracolumbar spine; motion between individual vertebra was not targeted. Statistical models were made on stride-by-stride data. As a sensitivity analysis eight variables with statistically significant but numerically (subjectively) small differences were rerun with trial-means data (n = 90 datapoints). The between-gait differences were then no longer significant for thoracolumbar lateral bending mean and pelvic yaw mean.

In the current study, minor asymmetries in neck and back motion patterns were observed both at group level and in individual horses. For comparison, mild vertical motion asymmetries are common in riding horses perceived sound by their owners, and their background is uncertain and likely multifactorial (Persson-Sjödin et al. 2019; Byström et al. 2020). However, a previous study of warmblood horses in trot found that cervical and thoracolumbar ROM varied 0.8–1° on average between measurements in individual horses (Hardeman et al. 2020). Further, another study noted that a 0.5–1.5° change in thoracolumbar lateral bending mean is not uncommon following removal and re-placement of markers, without a corresponding change in the relative difference between left and right directions (Byström et al. 2021). For these reasons, it is important not to overemphasise small offsets or differences between directions, particularly in individual horses and single measurements.

### Conclusions

When moving on a small circle horses show a relatively similar degree of cervicothoracic and thoracolumbar lateral bending to the inside in all three basic gaits, whereas cervicothoracic and thoracolumbar lateral bending ROM and pelvic yaw ROM were higher in walk and canter compared to trot. Data traces were often similar between horses with different conformation, even if minor individual differences could be seen. Taken together, the study findings suggest that cervicothoracic and thoracolumbar motion patterns on the circle reflect an interaction between the constraints of circular movement, and the mechanics and characteristics of each gait. Knowledge about the differences in back motion between different gaits, and how these differ between straight line and circle, can be helpful during clinical assessment of equine back motion and for achieving specific goals during training and rehabilitation.

### Supplementary information

Below is the link to the electronic supplementary material.

Supplementary file1 (DOCX 14 KB)

Supplementary file2 (DOCX 899 KB)

Supplementary file3 (DOCX 931 KB)

Supplementary file4 (XLSX 40 KB)
