# Supplementary material for: Back motion in unridden horses in walk, trot and canter on a circle
Source: Vet Res Commun. 2023 May 2;47(4):1831–43. doi: 10.1007/s11259-023-10132-y (PMC10698108; doi:10.1007/s11259-023-10132-y)
Supplement: Supplementary file 1 — Supplementary file1 (DOCX 14 KB) [file 11259_2023_10132_MOESM1_ESM.docx]

Veterinary Research Communications

Back motion in unridden horses in walk, trot and canter on a circle

Agneta Egenvall^1^,* Hanna Engström^2^, Anna Byström^3^

1. Department of Clinical Sciences, Faculty of Veterinary Medicine and Animal Science, Swedish University of Agricultural Sciences, Uppsala, Sweden; agneta.egenvall@slu.se; 0000-0002-8677-6066

2. Ekeskogs Riding Academy, Klintehamn, Sweden; ekeskogs@gmail.com;

3. Department of Anatomy, Physiology and Biochemistry, Faculty of Veterinary Medicine and Animal Science, Swedish University of Agricultural Sciences, Uppsala, Sweden; anna.bystrom@slu.se; 0000-0002-2008-8244

*Correspondence: agneta.egenvall@slu.se; Tel.: (+46-703799544)

S1 Table . Characteristics of the 16 horses in the study.

| Horse | Breed | Gender | Age  (years) | Height at  withers (cm) | Duration with  owner (years) |
| --- | --- | --- | --- | --- | --- |
| A | PRE^a^ | Stallion | 24 | 155 | 5 |
| B | American Curly | Mare | 17 | 153 | 6 |
| C | Russian crossbred /PRE | Gelding | 17 | 158 | 3 |
| D | Swedish warmblood | Gelding | 19 | 167 | 4 |
| E | Iberian cross | Mare | 4 | 143 | 4 |
| F | Lusitano | Gelding | 11 | 150 | 2 |
| H | Swedish warmblood/PRE | Mare | 14 | 158 | 9 |
| I | PRE | Stallion | 11 | 153 | 10 |
| J | Friesian | Mare | 9 | 158 | 5 |
| M | New Forest | Gelding | 10 | 141 | 5 |
| P | PRE | Gelding | 11 | 155 | 3 |
| Q | PRE | Mare | 8 | 157 | 5 |
| S | Irish Cob | Gelding | 16 | 152 | 6 |
| V | Lusitano | Mare | 16 | 154 | 10 |
| X | Iberian cross | Gelding | 8 | 165 | 8 |
| Y | PRE | Gelding | 9 | 158 | 9 |

^a^PRE-Pura Raza Espanol
