# Supplementary material for: Back motion in unridden horses in walk, trot and canter on a circle
Source: Vet Res Commun. 2023 May 2;47(4):1831–43. doi: 10.1007/s11259-023-10132-y (PMC10698108; doi:10.1007/s11259-023-10132-y)

Veterinary Research Communications

Back motion in unridden horses in walk, trot and canter on a circle

Agneta Egenvall^1^,* Hanna Engström^2^, Anna Byström^3^

1. Department of Clinical Sciences, Faculty of Veterinary Medicine and Animal Science, Swedish University of Agricultural Sciences, Uppsala, Sweden; agneta.egenvall@slu.se; 0000-0002-8677-6066

2. Ekeskogs Riding Academy, Klintehamn, Sweden; ekeskogs@gmail.com;

3. Department of Anatomy, Physiology and Biochemistry, Faculty of Veterinary Medicine and Animal Science, Swedish University of Agricultural Sciences, Uppsala, Sweden; anna.bystrom@slu.se; 0000-0002-2008-8244

*Correspondence: agneta.egenvall@slu.se; Tel.: (+46-703799544)


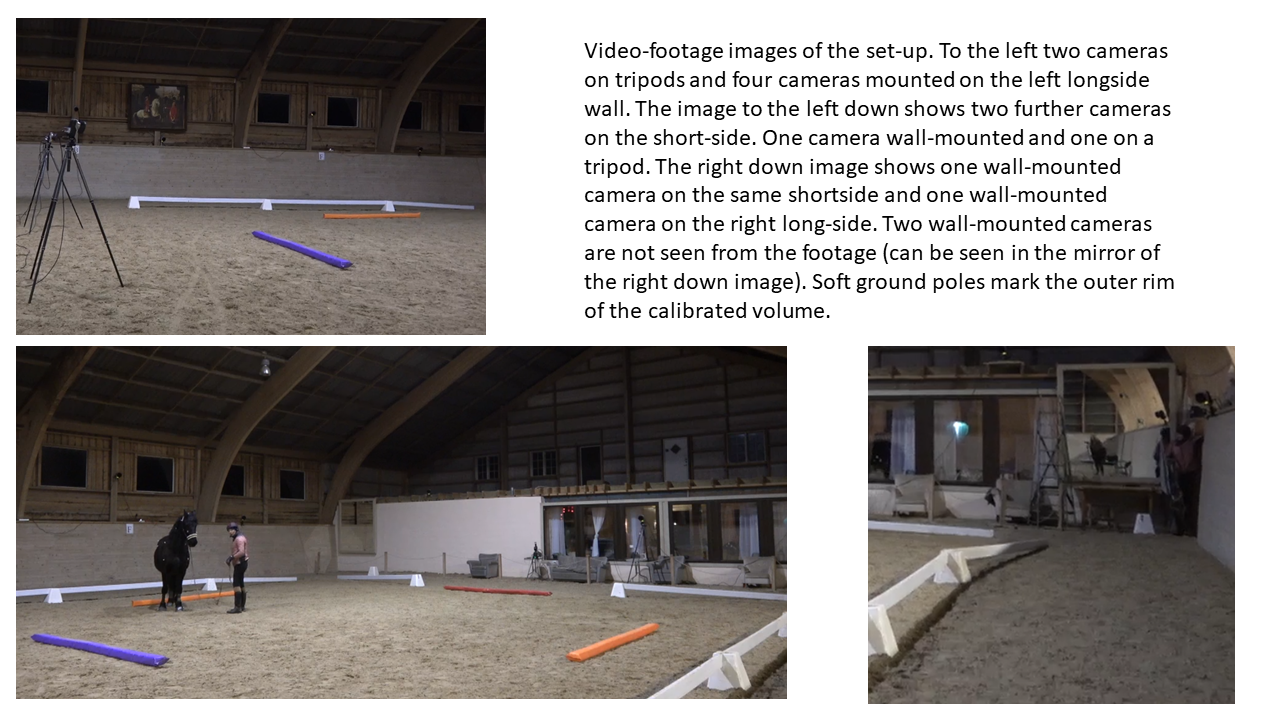

Supplement: Supplementary file 2 — Supplementary file2 (DOCX 899 KB) [file 11259_2023_10132_MOESM2_ESM.docx]
