# Supplementary material for: Back motion in unridden horses in walk, trot and canter on a circle
Source: Vet Res Commun. 2023 May 2;47(4):1831–43. doi: 10.1007/s11259-023-10132-y (PMC10698108; doi:10.1007/s11259-023-10132-y)

Veterinary Research Communications

Back motion in unridden horses in walk, trot and canter on a circle

Agneta Egenvall^1^,* Hanna Engström^2^, Anna Byström^3^

1. Department of Clinical Sciences, Faculty of Veterinary Medicine and Animal Science, Swedish University of Agricultural Sciences, Uppsala, Sweden; agneta.egenvall@slu.se; 0000-0002-8677-6066

2. Ekeskogs Riding Academy, Klintehamn, Sweden; ekeskogs@gmail.com;

3. Department of Anatomy, Physiology and Biochemistry, Faculty of Veterinary Medicine and Animal Science, Swedish University of Agricultural Sciences, Uppsala, Sweden; anna.bystrom@slu.se; 0000-0002-2008-8244

*Correspondence: agneta.egenvall@slu.se; Tel.: (+46-703799544)

Left (red) and right (blue) directions are plotted superimposed; the thick lines represent average across strides, and the shading between-stride variation. The stride starts and ends at inner hind limb maximum protraction. The patterns clearly differ between gaits for both horses, but is generally similar between directions. Comparing the same gait between the two horses, both similarities and the differences can be identified.

S3 Figure 1. Time-normalised stride data by direction: left (red) and right (blue) in walk, trot and canter for horse D. The x-scale is in percent of the stride relative to inside hind limb maximum protraction. Thick lines represent mean values across all available strides, and the surrounding shaded area SD. The curves are based on between 13 and 24 strides. Lateral bending angles were defined as negative for bending to the left and positive for bending to the right. For definition of pelvic rotations see Fig 3. The trunk horizontal angle was defined as positive for ‘forehand to the right - hind quarters to the left’ deviation relative to the direction of motion, and the neck-to trunk angle was positive if the head was placed to the right relative to the orientation of the body.


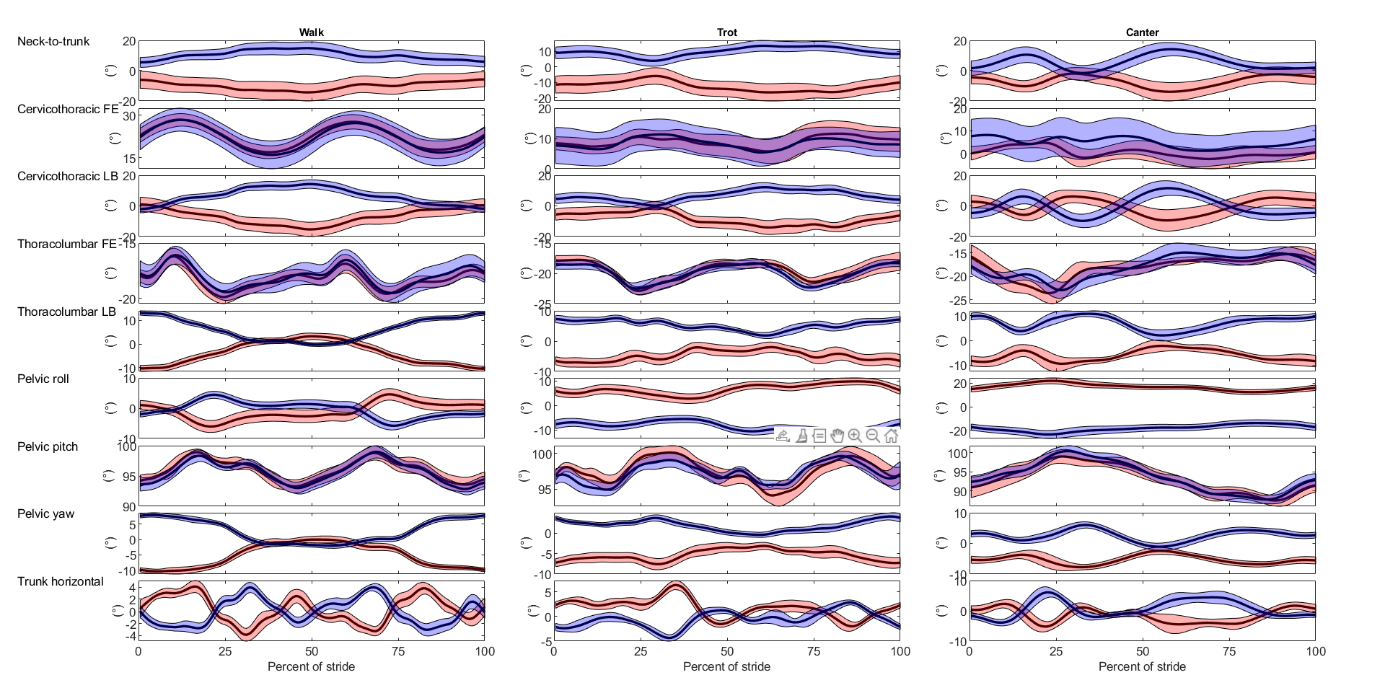


S3 Figure 2. Time-normalised stride data by direction: left (red) and right (blue) in walk, trot and canter for horse S. The x-scale is in percent of the stride relative to inside hind limb maximum protraction. Thick lines represent mean values across all available strides, and the surrounding shaded area SD. The curves are based on between 9 and 23 strides. Lateral bending angles were defined as negative for bending to the left and positive for bending to the right. For definition of pelvic rotations see Fig 3. The trunk horizontal angle was defined as positive for ‘forehand to the right - hind quarters to the left’ deviation relative to the direction of motion, and the neck-to trunk angle was positive if the head was placed to the right relative to the orientation of the body.


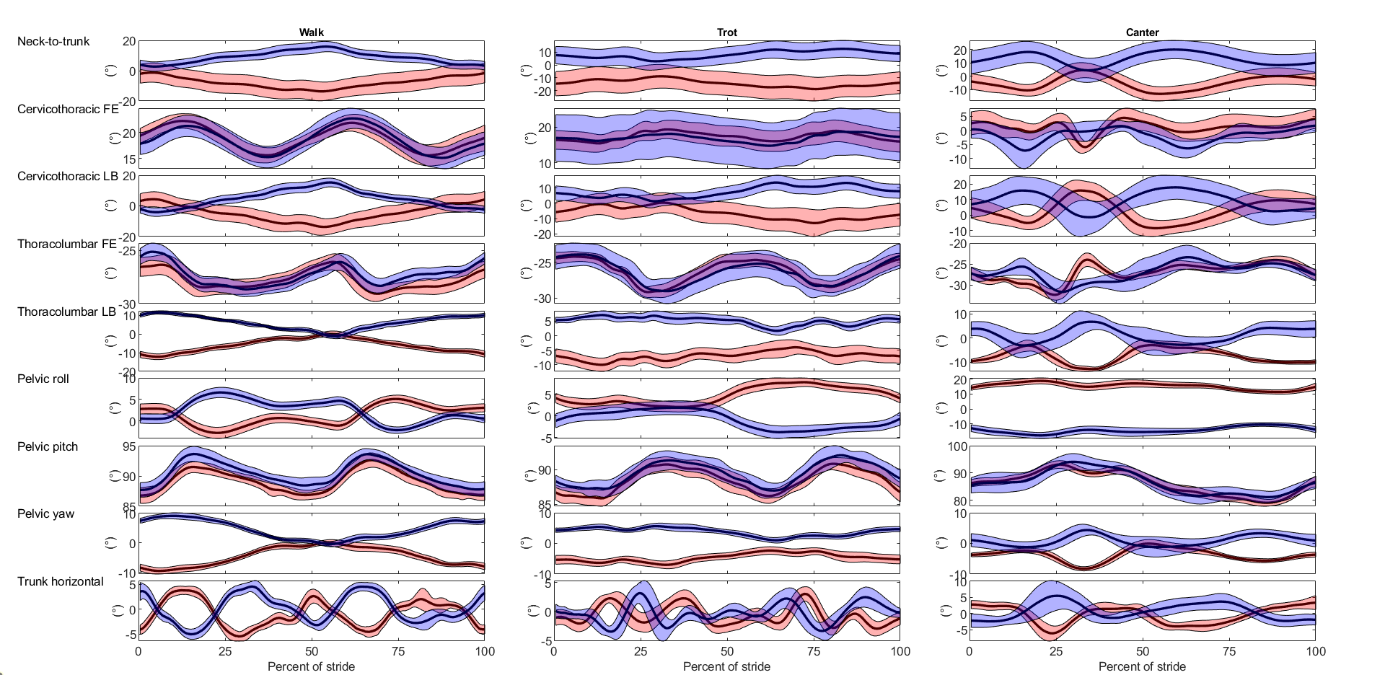

Supplement: Supplementary file 3 — Supplementary file3 (DOCX 931 KB) [file 11259_2023_10132_MOESM3_ESM.docx]
